# Pokeweed Antiviral Protein, a Ribosome Inactivating Protein: Activity, Inhibition and Prospects

**DOI:** 10.3390/toxins7020274

**Published:** 2015-01-28

**Authors:** Artem V. Domashevskiy, Dixie J. Goss

**Affiliations:** 1 John Jay College of Criminal Justice, Department of Sciences, City University of New York, 524 West 59th Street, New York, NY 10019, USA; 2 Department of Chemistry, Hunter College, City University of New York and the Graduate Center, 695 Park Avenue, New York, NY 10065, USA; E-Mail: dgoss@hunter.cuny.edu

**Keywords:** pokeweed antiviral protein, ribosome inactivating protein, virus genome-linked protein, sarcin/ricin loop, immunotoxin

## Abstract

Viruses employ an array of elaborate strategies to overcome plant defense mechanisms and must adapt to the requirements of the host translational systems. Pokeweed antiviral protein (PAP) from *Phytolacca americana* is a ribosome inactivating protein (RIP) and is an RNA *N*-glycosidase that removes specific purine residues from the sarcin/ricin (S/R) loop of large rRNA, arresting protein synthesis at the translocation step. PAP is thought to play an important role in the plant’s defense mechanism against foreign pathogens. This review focuses on the structure, function, and the relationship of PAP to other RIPs, discusses molecular aspects of PAP antiviral activity, the novel inhibition of this plant toxin by a virus counteraction—a peptide linked to the viral genome (VPg), and possible applications of RIP-conjugated immunotoxins in cancer therapeutics.

## 1. Introduction

An evolutionary arms race between plants and their pathogens has shaped each other’s elaborate strategies for survival. Many plants produce toxic proteins that are thought to play a key role in their defense mechanisms against foreign pathogenic invaders. These anti-pathogenic protein toxins are known as ribosome inactivating proteins (RIPs). RIPs are broadly distributed throughout the kingdom of plants, fungi, and have been identified in several species of bacteria. High toxicity of the castor (*Ricinus communis*) and jequirity (*Abrus precatorius*) bean plants owe their detrimental physiological effects toward eukaryotic cells to these poisons and have been known since antiquity [1]. The deadliness of many RIPs has been explored by political and military organizations to design biological weaponry [2,3,4], many scientists to generate transgenic species of plants resistant to viral and fungal infections [5,6], numerous cancer researchers in production of immuno-conjugate therapeutics [7,8,9], as well as mystery writers to engage the readers [10]. RIPs are RNA *N*-glycosidases that inhibit advanced stages of protein synthesis by selectively modifying large rRNA molecules and deactivating ribosomes [11]. Other plants (e.g., common pokeweed—*Phytolacca americana* and common soapwart—*Saponaria officinalis*) produce pokeweed antiviral protein (PAP) [12] and saporin [13], respectively, with increased antiviral and antifungal activities. Presently, evidence for the lack of RIPs has been obtained solely for *Arabidopsis thaliana*, as this plant does not express detectible amounts of RIPs nor contains a sequence that encodes for any putative RIP in its genome [14]. Generally, RIPs being potent cellular toxins are exported out of the cell once they are synthesized, and localized within the cell wall matrix [15]. It is hypothesized that they gain access into the cytoplasm as the pathogen enters the cell, thus promoting their activity by impairing host ribosomes [16].

## 2. Pokeweed Antiviral Protein: One of a Number of Ribosome Inactivating Proteins

### 2.1. Introduction to the Ribosome Inactivating Proteins

The term “ribosome inactivating protein” came about before the structure and enzymatic activities of RIPs were realized. After the mechanism of action of RIPs on ribosomes became clear, the name is used for these *N*-glycosidases [EC 3.2.2.22]. Enzymes known as proteases and RNases inactivate or damage ribosomes by different mechanisms, and may not be classified as RIPs [17].

#### 2.1.1. Classification of Pokeweed Antiviral Protein among Other Ribosome Inactivating Proteins

The first acknowledged RIPs were ricin and abrin [18,19]. However, only in 1971 ricin was recognized as an inhibitor of eukaryotic protein synthesis [20]. It was not established until several years later that the inhibition of protein synthesis was due to the impairment of host ribosomes [21]. PAP was also reported to impair protein synthesis through a related mechanism [22].

**Figure 1:**
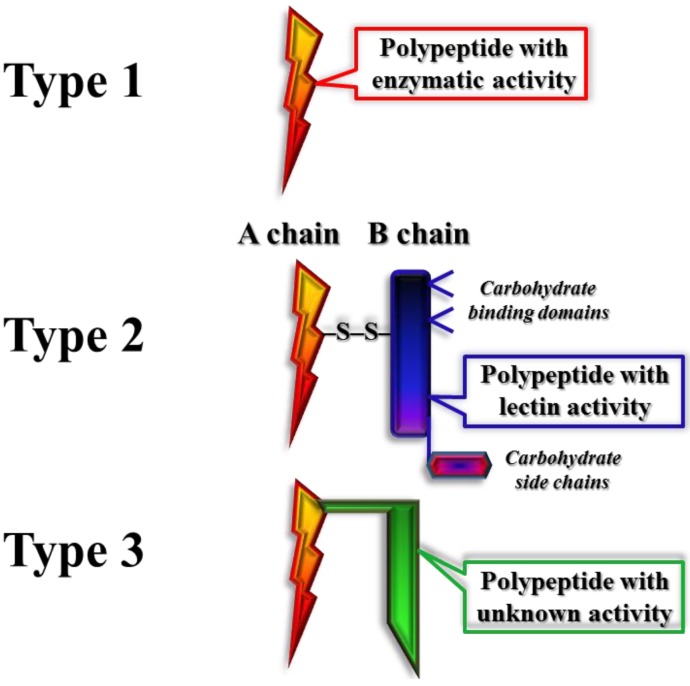
Schematic depiction of the structure of ribosome inactivating proteins (RIPs).

Classification of RIPs into holo- and chimero-subgroups was founded on their physical properties, the number of polypeptide chains, and posttranslational modifications (Figure 1) [23,24]. Holo-RIPs, having a single RNA *N*-glycosidase domain, are frequently referred to as type 1 RIPs. These consist of a single intact polypeptide of ~30 kDa [12,25]. Type 1 RIPs are strongly basic proteins that are clearly distinct in their global sequence homology and posttranslational alterations, yet share several active site residues and secondary structure elements [26,27,28]. Examples of type 1 RIPs include PAP, saporin, and barley (*Hordeum vulgare*) translational inhibitor. Type 1 RIPs inhibit cell-free protein synthesis and are only mildly toxic to cells and animals. The majority of characterized RIPs fall into this category [25]. Among type 1 RIPs, the 3D structures of saporin, momordin (MOM), PAP, trichosanthin and gelonin have been determined and their properties characterized [26,29,30,31,32]. Fifteen isoforms of saporin from *S. officinalis* have been characterized, differing in ribosome translation inhibition activities and cellular toxicity [33]. Crystal structure at 2.0 Å resolution of isoform 6 of saporin (SO6) has been reported with a structural motif that includes three lysyl residues in its *C*-terminal region—a highly conserved motif in all RIPs [29]. X-ray crystal structure of PAP has been determined [34] with various active site inhibitors and rRNA substrate analogues. Graphical Abstract presents a low temperature structure of PAP (PDB ID 1QCI).

Chimero-RIPs contain two structurally and functionally distinct domains: the catalytic RNA *N*-glycosidase domain and the carbohydrate (lectin properties) binding domain, and are better known as type 2 RIPs. Examples of these acutely toxic heterodimeric proteins include ricin, abrin and modeccin. Their amino-terminal domain is equivalent to the catalytic domain of type 1 RIPs—the RIP activity domain, often referred to as the A-chain (e.g., RTA—ricin A-chain). The catalytic domain is disulfide bridge-linked to an evolutionary divergent carboxyl-terminal B-chain, possessing sugar-binding properties of ~30 kDa [35,36,37]. Galactosyl moieties of glycoproteins and/or glycolipids, localized on the exterior of eukaryotic cells [38,39,40], are bound by the lectin chains of type 2 RIPs. This binding promotes the reverse transport of the A-chain to the cytosol [41,42,43]. Once inside the cell, the RIP accesses translational machinery and depurinates ribosomes. Extracellular location of RIPs prevents contact between these poisons and the ribosomes of healthy cells, yet provides an immediate source of the toxin when a pathogen infects the cell. The type 2 RIPs have proven to be invaluable for studies of endocytosis and intracellular transport into mammalian cells [44,45,46]. It is not clear how type 1 RIPs are distributed within the extra-cellular spaces of the host cells, although primary structure analysis points to fatty acid binding sequences within their sequences [15,47]. Clear evidence however, has been presented for PAP retro-translocation from the endoplasmic reticulum into the cytosol [48].

Type 3 RIPs are synthesized as inert precursors (pro-RIPs), and undergo proteolytic modifications to acquire their enzymatically activity [23]. These RIPs are much less common than either type 1 or type 2 RIPs. Type 3 RIPs have been identified from maize (*Zea mays*) and barley (from *H. vulgare*) [49,50,51,52].

#### 2.1.2. Distribution of Ribosome Inactivating Proteins

RIPs are broadly dispersed among plants, fungi, alga, and several species of bacteria [25,53,54]. Additionally, RIP-type activity has been reported in animal tissues as well [55]. A large number of RIPs has been identified in a small group of families, namely *Carylphyllaceae*, *Cucurbitaceae*, *Euphorbiaceae*, *Sambucaceae*, *Phytolaccaceae* and *Poaceae* [24]. Synthesis of some RIPs could be induced by factors, such as senescence [56], viral infection [57], development [51] and stress [58].

The molecular weight of type 1 RIPs ranges within 21–38 kDa. As for type 2 RIPs, the molecular weights of the two-chain peptide range from 56 to 69 kDa [24]. Bacterial RIPs Stx1 and Stx2 from *Escherichia coli* (*E. coli*) promote their enzymatic activity similar to their plant analogues [59,60,61,62,63]. Research reveals that RIPs are found in several fungi species [64,65,66,67]. At least one RIP has been isolated from alga, *Laminaria japonica* A. [68]. All of the above findings favor the generally accepted hypothesis that RIPs are enzymes widely distributed in nature, and therefore play pivotal undefined biological roles.

##### Pokeweed Antiviral Protein and Its Isoforms

Most type 1 RIPs are encoded by intron-less genes that define pro-RIPs with *N*- and *C*-terminal extensions with respect to the mature forms, e.g., several isoforms of pokeweed antiviral protein from *P. americana* have been described (Table 1) [12,24,69,70,71]. All of them possess pronounced antiviral properties and high enzymatic activity on ribosomes from diverse phyla. These isoforms are encoded by a gene family composed of approximately nine members [69]. PAPI (or simply PAP), PAP-II and PAP-III are the leaf isoforms that appear in spring, early and late summer, respectively [12,69,70,71,72,73], whereas PAP-S1 and PAP-S2 are the isoforms isolated from seeds and have been shown to exhibit the highest activity *in vitro* of all the isoforms [74,75,76]. PAP and PAP-S1 share 76% sequence identity, PAP-S1 and PAP-S2 have 83%, whereas PAP and PAP-II are only 33% identical [76,77]. A further isoform, α-PAP, is similar in sequence to PAP-S1, and essentially expressed in all organs [76,77]; it shares 74% identity with PAP. PAP-R has been isolated from roots of the pokeweed plant [24,78] and PAP-H is from hairy roots [24,79]. Moreover, RIP-free callus and suspension cultures of *P. americana* have been attained [24,80]. Perhaps, a gene-silencing event occurred during the establishment of the cultures because RIP-isoforms are ubiquitously expressed in all organs of the plant [77].

The genes of PAP [69], PAP-II [72] and PAP-S [81] have been isolated from tissue specific cDNA libraries and sequenced. The PAP gene carries an open reading frame of 939 nt coding for the mature PAP protein (262 amino acids) plus an *N*-terminal signal peptide of 22 amino acids [69] and a *C*-terminal extra peptide of 29 amino acids [30]. This gene has been expressed in *E. coli* cells under an inducible (*lac*) promoter with an extremely low yield (0.13%–0.16% of the total bacterial protein) [82]. It was found that even the low level of gene expression slowed down bacterial growth significantly. Chen *et al.*, also found that elimination of *N*-terminal signal peptide codons (22 amino acids) from the PAP gene led to an immediate cell death [82]. The authors have concluded that PAP is highly toxic (*in vivo*) for prokaryotic cells [83]. Crystal structure of PAP-I, α-PAP, and PAP-II have been determined at different resolutions [30,34,84]. PAP-S differs from other PAP isoforms; it is associated with three *N*-acetylglucosamine residues covalently attached to the protein’s asparagine residues [85]. Based on the X-ray and molecular modeling studies, PAP-III is predicted to have a greater anti-HIV activity due to its topology and charge distribution [86]. Moreover, modeling studies have indicated that PAP is able to accommodate a guanine base in its active pocket without large conformational changes, and this prediction was experimentally confirmed [87].

**Table 1:** Isoforms of Pokeweed Antiviral Protein [24]. ND: Not Determined.

Isoform	Source	Number of Aminoacyl Residues	MW (kDa), Mature Protein	Activity (RC_50_)	References
PAP-I	Spring Leaves	262	29	1.5 nM Rat Liver Ribosomes; 4.7 nM *E. coli* Ribosomes	[70]
PAP-II	Early Summer Leaves	285	30	ND	[70]
PAP-III	Late Summer Leaves	285	30	ND	[86,88]
PAP-S1	Seeds	262	29	3.2 nM Rat Liver Ribosomes; 280 nM *E. coli* Ribosomes	[75,76]
PAP-S2	Seeds	262	29	3.6 nM Rat Liver Ribosomes; 1000 nM *E. coli* Ribosomes	[75,76]
α-PAP	Expressed in All Organs	261	28.9	1.3 nM Rat Liver Ribosomes; 25 nM *E. coli* Ribosomes	[76]
PAP-R	Roots	271	29.8	ND	[89]
PAP-H	Hairy Roots	268	29.5	ND	[90]
PAP-Culture	Tissue Culture	262	29	ND	[91]

### 2.2. Biological and Enzymatic Activities of Ribosome Inactivating Proteins

Distinct biological activity of both type 1 and type 2 RIPs has served as the basis for their identification. Type 2 RIPs owe their toxicity and cytotoxicity to the deviations in the lectin activity and specificity of the B-chain, and present with significant differences in their cytotoxicity. Ricin, for instance, is known to induce 50% apoptosis in cells at concentrations below 1 ng/mL, while some elderberry type 2 RIPs display no significant effects at 1 mg/mL [78].

The inhibitory effect of PAP on tobacco mosaic virus (TMV) transmission was reported in 1925 by Duggar and Armstrong [79], yet PAP was not acknowledged as a protein synthesis inhibitor until 1978 [80]. Myriad type 1 RIPs are antiviral proteins. Type 1 RIPs are not as cytotoxic as type 2 RIPs, since they are not able to cross the cell membrane on their own. Nevertheless, a number of specialized animal cells are able to import type 1 RIPs by endocytosis, and consequently are susceptible to the RIP activity.

Monomeric protein synthesis inhibitors share a significant sequence identity with ricin’s A-chain (RTA). Further, ricin, abrin, and PAP inhibit cell-free protein synthesis by permanent inactivation of the ribosomes by means of arresting the function of elongation factors EF-1 and EF-2 [25,92].

**Figure 2:**
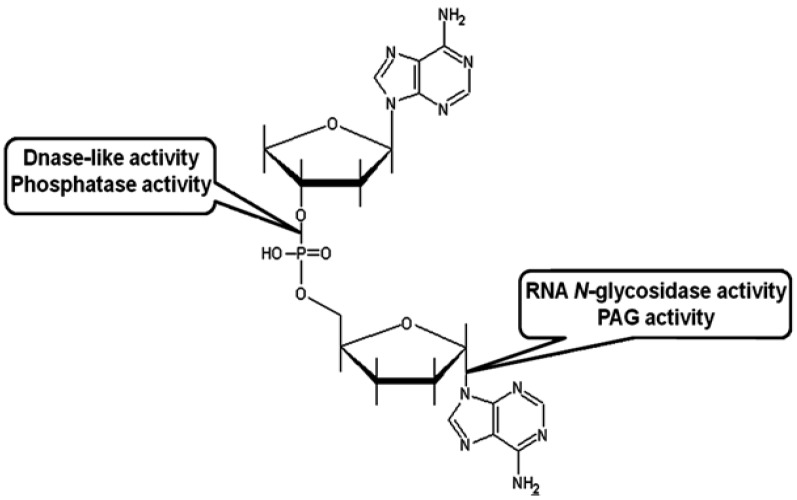
Schematic representation of the action sites for RNA *N*-glycosidase activity, polynucleotide:adenosine glycosidase (PAG) activity, and presumed DNase-like and phosphatase activity of RIPs.

It is well known that some RIPs possess multiple enzymatic activities. Some RIPs act on rRNA at specific single sites [93]; yet others were shown to depurinate multiple adenines from various nucleic acid substrates, such as herring sperm DNA, poly(A), tRNA, and even TMV RNA [94]. Site-specific RNA *N*-glycosidase activity (Figure 2) toward ribosomes, rRNA, and depurination of mRNA and viral RNA are briefly discussed below.

#### 2.2.1. Site-Specific RNA *N*-Glycosidase Activity towards Ribosomes and Naked rRNA

**Figure 3:**
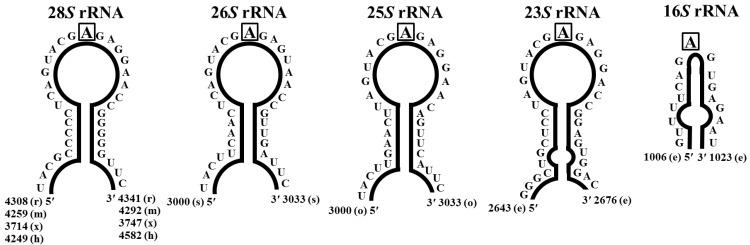
Structure of rRNA substrates for *N*-glycosidase activity of RIPs: (e) *E. coli*; (h) *Homo sapiens*; (m) *Mus musculus*; (o) *Oryza sativa*; (r) *Rattus rattus*; (s) *Saccharomyces cerevisiae*; (x) *Xenopus laevis*.

Ricin and other RIPs recognize a specific and universally conserved region within the large 28S rRNA, and cleave a glycosidic bond between an adenine and the nucleotide on the RNA [95]. For rat liver ribosome, this distinct site is A4324, and it is positioned within a single-stranded loop referred to as the sarcin/ricin (S/R) loop. The S/R loop is located within the domain VII of the 28S rRNA (Figure 3) [25,96]. After the adenine is removed, the depurinated site becomes subject to a β-elimination hydrolysis when treated with acidic aniline. This promotes cleavage of the 3'-end of the rRNA, and the depurination product can be detected by electrophoresis. This site-specific RNA *N*-glycosidase activity is a common characteristic attributed to all RIPs. Schramm *et al.*, showed that the transition state of the ricin reaction develops an oxocarbenium character on the ribose [97]. Furthermore, it was established that RTA operates via a D_N*_A_N_ mechanism [98]. This was further confirmed by the synthesis of novel compounds that incorporated the cationic character, such as amines, into a ribose analogue. As expected for true transition state analogues, these were potent, tightly binding, ricin inhibitors [98]. Tanaka *et al.*, have used the RTA transition state knowledge to design and synthesize a high affinity inhibitor of the RTA catalytic site [98]. PAP and other RIPs inhibit the translocation step of elongation [99]. Specifically, PAP inhibits Ty*1*-directed +1 ribosomal frameshifting and retrotransposition [100,101].

The S/R loop within the rRNA of different ribosomes is universally conserved across all species (Figure 3) [24]. Consequently, the specificity of different RIPs for their substrates, and the sensitivity between ribosomes among species are likely to come from deviations within RIPs themselves and the ribosomal proteins. Ricin presents highest activity toward yeast and mammalian ribosomes, but exhibits low activity on plant and *E. coli* ribosomes [25], whereas PAP depurinates ribosomes from plants, bacteria, yeasts, and lower and higher animals [25]. RIP substrate accessibility may be influenced by the deviations in ribosomal proteins that would dictate RIP activity and sensitivity towards different ribosomes. The L9 and L10e rat liver ribosomal proteins are targeted by the ricin A chain (RTA) [102], however PAP has been shown to bind to the L3 yeast ribosomal protein [103,104]. Transgenic plants, expressing truncated L3 ribosomal protein, confer resistance to PAP [104]. Furthermore, broad-spectrum activity of PAP towards different ribosomes may be explained by the fact that the L3 ribosomal protein is highly conserved. Pokeweed ribosomes were shown to be as sensitive to PAP treatment as wheat ribosomes [16]. The rRNA in native ribosomes is the ideal substrate for RIPs, nonetheless, protein-free rRNA [105] and synthetic oligoribonucleotides that mimic the S/R domain [106,107], serve as substrates for RIP activity as well. All RIP depurinate the equivalent adenine residue from naked rRNA as from native ribosomes, but many of them depurinate naked rRNA at multiple sites. In addition, several RIPs are able to depurinate naked rRNA from non-substrate ribosomes. For instance, ricin is able to act on naked *E. coli* 23*S* rRNA, however possesses no activity against the intact *E. coli* ribosomes. Moreover, several RIPs are able of depurinating guanine residues within their substrates [34,108].

#### 2.2.2. Depurination of Capped and Uncapped mRNA; Antiviral Action of PAP

Advances in high-performance liquid chromatography (HPLC) coupled to fluorescent methods of detection, allowed researchers to detect, identify, and quantify possible reaction products of RIPs and the amount of free adenine released from different substrates by RIPs [107,109]. These techniques allow for direct measurement of ribosomal depurination, quantification of released purines, and also aided in identification of some uncommon activities of RIPs. For instance, various RIPs serve as effective inhibitors of animal and/or plant viruses. Depurination of viral RNA by RIPs is a novel activity, and any insights into the mechanism of substrate selectivity and specificity may be of paramount importance in the search for the potent antiviral agents. The mode of action for the antiviral activity of RIPs is poorly understood, however this activity does not depend solely on the ribosomal inactivation. An alternative mechanism may involve a direct interaction of RIP with viral RNA or DNA, with additional effects brought about by the eukaryotic translation initiation factors (eIFs). Pokeweed antiviral proteins PAP-I, PAP-II, and PAP-III cause a concentration-dependent depurination of genomic HIV-1 RNA [88,110,111], TMV RNA [112], poliovirus [113], herpes simplex virus [114], influenza virus [115], brome mosaic virus (BMV) [116], lymphocytic choriomeningitis virus (LCMV) [117], tobacco etch virus (TEV) RNA [107], and inhibits Japanese encephalitis virus infection, both *in vitro* and *in vivo* [118]. In contrast, the RTA does not depurinate same viral RNAs to produce detectable quantities of purines.

A novel mechanism for the inhibition of translation by PAP has been put forward [119]. This inhibition of translation is based on a specific depurination of capped mRNA. Hudak *et al.*, used wild type (WT) PAP and three different PAP mutants (PAPx, an active site mutant (E176V); PAPn, a mutant with a substitution (G75D) in the amino-terminal sequence; PAPc, a mutant lacking the carboxyl-terminal 25 amino acid residues) that do not depurinate tobacco or rabbit ribosomes, and have shown that PAP inhibits the *in vitro* translation of BMV and potato virus X RNAs without ribosomal depurination [119]. This shows that PAP is able to differentiate between capped and uncapped mRNAs, since PAP, and some of its mutants, inhibited the translation of capped (but not uncapped) luciferase transcripts. Presence of m^7^GTP analogue lowers translational inactivation activity of PAP and PAP mutants, implying that these RIPs are able to recognize the cap structure on the mRNAs [120]. PAP-treated luciferase transcripts revealed that the capped, but not the uncapped RNAs were subject to degradation by acidic aniline, and therefore were depurinated *in vitro*. It was concluded that PAP may inhibit translation by binding to the cap structure and depurinating the RNA, and that depurination of capped viral RNA may be the principal mechanism for the antiviral activity of PAP [120]. Baldwin *et al.* [121] have characterized the interactions of PAP with m^7^GTP cap analogue using fluorescence spectroscopy, and these interactions were previously identified as competitive [107]. Zoubenko *et al.*, have presented evidence that PAP depurinates tobacco ribosomes *in vivo* by removing more than one adenine and a guanine [122]. Moreover, PAP_n_ mutant did not bind ribosomes efficiently pointing to the importance of Gly-75 for PAP to bind ribosomes. Unlike wild type PAP (or the *C*-terminal PAP_C_ mutant), PAP_n_ did not trigger production of salicylic acid in transgenic plants [122].

These results depict a promising mechanism to explain the antiviral activity of PAP, however, some queries remain. For instance, the above-proposed mechanism does not clarify the inhibitory effect of PAP on the replication of uncapped viruses such as influenza [115] and poliovirus [113]. Vivanco *et al.*, [123] have examined the activity of PAP against a variety of capped and uncapped viral RNAs, and demonstrated that PAP does not depurinate every capped RNA, and that it can inhibit translation of uncapped viral RNAs *in vitro* without causing detectable depurination. PAP depurinated the capped TMV and BMV RNAs, but did not depurinate the uncapped luciferase RNA, indicating that PAP can distinguish between capped and uncapped RNAs, but no detectable depurination of capped alfalfa mosaic virus (AMV) RNA was recorded. This implies that recognition of the cap structure alone is not sufficient for depurination of RNAs [123]. Moreover, PAP did not cause detectable depurination of uncapped RNAs from tomato bushy stunt virus (TBSV), satellite panicum mosaic virus (SPMV), and uncapped RNA containing poliovirus IRES (internal ribosome entry site); however, *in vitro* translation experiments showed that PAP inhibited translation of the above viral RNAs [123].

#### 2.2.3. Effects of eIFs and RNA Secondary Structure—Missing Links in PAP-Substrate Selectivity

Wang *et al.* [124] presented evidence that PAP binds to eIF4G and its isoform eIF*iso*4G. In wheat (*Triticum aestivum*), two forms of eIF4G exist, which differ in size, 180 (eIF4G) and 86 kDa (eIF*iso*4G), and they bear only 30% amino acid identity [125,126]. PAP binds to m^7^GTP-Sepharose and this interaction does not diminish the binding of PAP to purified eIF*iso*4G, indicating that a complex can form between the cap structure, PAP and eIF*iso*4G. In the presence of wheat germ lysate, PAP depurinated uncapped transcripts having a functional WT 3'translational enhancer sequence (3'TE), but did not depurinate messages containing a non-functional mutant 3'TE [124]. This result supports earlier hypothesis that binding of PAP to eIF4G and eIF*iso*4G could provide a mechanism for PAP to access both uncapped and capped viral RNAs for depurination. In support to the above findings, Baldwin *et al.* [121] have shown that PAP not only binds to the initiation factor scaffolding protein eIF*iso*4G, but that binding of cap analogue to PAP is increased by these protein-protein interactions, suggesting a model where PAP interacts with eIF*iso*4G/eIF4G (as part of the eIF*iso*4F/eIF4F complex) and binds to the cap region of mRNA. Furthermore, addition of eIF*iso*4E/eIF4E (as part of the eIF*iso*4F/eIF4F complex) lowers the binding affinity of PAP for the cap competitively because both are specific cap-binding proteins. The ability of PAP to lower infectivity of both capped and uncapped RNA viruses suggests the presence of a different, other than m^7^G cap, requirement that may influence PAP substrate recognition, binding, and its antiviral activity. This was further supported by the pull-down assay and the Fluorescence Resonance Energy Transfer (FRET) experiments, where Cheng *et al.* showed that the formation of a triplex complex between PAP, eIF*iso*4E and eIF*iso*4G dramatically increase FRET energy transfer upon binding of the eIF*iso*4G to the binary PAP-eIF*iso*4E complex [127]. This triplex protein interaction demonstrates that eIF*iso*4G plays a key role in the regulation of PAP binding.

Elements within RNA secondary structures have been identified to play differentiated roles in PAP binding to various structured RNAs. The 3'-UTRs of the non-polyadenylated plant viral mRNAs of TMV and BMV are known to increase both the stability and the translational efficiency of a message [128,129] in carrot protoplasts, whereas those of turnip mosaic (TYMV) and AMV viruses show no (or lowered) effect on gene expression [129]. PAP depurinating activity on these structural viral RNAs correlates to the 3'-UTRs translational effect [130]. Moreover, the presence of the eIFs reveal drastic differences in the activity of PAP for its viral substrates, in a way that eIF*iso*4F (complex of eIF*iso*4E and eIF*iso*4G) promotes increased affinity (and activity) of PAP for the TMV and BMV RNAs, whereas there is a negligible effect of eIF*iso*4F on the binding to the TYMV and AMV, and no profound effect on the depurination of the these RNAs [131]. These PAP-eIF-RNA interactions possibly promote PAP active site structural changes, allowing PAP to recognize purine residues for depurination.

### 2.3. Physiological Role, Toxicity of RIPs and Immunotoxins

#### 2.3.1. Physiological Role of RIPs

Presently, there is no unequivocal and agreeable answer to why plants produce and accumulate RIPs, despite the comprehensive knowledge of their structure, activity, and action mechanism. RIPs are synthesized in many, but not all, plant species. Sequencing of the genome of *A. thaliana* provided no evidence of RIP encoding genes [14]. This suggests that RIPs are not ubiquitous among plant species, and do not play a universal role in their growth, development, or defense. Some facts support the notion that RIPs play a defense function in plants. Only type 2 RIPs are able to gain entree to the cytoplasm of intact cells via a receptor-lectin-mediated uptake process [25,132]. Toxicity of type 2 RIPs is restricted to animal cells because bacteria and fungi are protected by a cell wall; type 2 RIPs they must bind glycan receptors on the cell surface to ensure their entry. Ricin and abrin are thought to protect the seeds of these plants against plant-eating animals [133]. Type 1 RIPs have direct effect on yeast and plant pathogenic fungi [134]. Recent studies show that transgenic tobacco plant lines (*Nicotiana tabacum*), expressing an activated form of maize (*Z. mays*), a type 3 RIP, appear more resistant to larvae of the cigarette beetle (*Lasioderma Serricorne*) and the tobacco hornworm (*Manduca Sexta*) than the wild type plants [135], providing a resistance to these maize-eating insects. Presently though, there is no documented oral toxicity of type 1 and 3 RIPs on higher animals. RIPs possess a set of unique biological activities toward human and animal cells that could be exploited in antiviral drug therapeutics. The antiviral activity of type 1 RIPs is well documented [136], although the underlying mechanism has not been elucidated.

#### 2.3.2. Toxicity of Ribosome Inactivating Proteins

Reports about the use of ricin and abrin for homicidal purposes go back to ancient times. Nonetheless, usage of these toxins as regular weapons is a quite modern idea. The ease to acquire large amount of ricin, for example, made this toxin a good candidate for bioterrorism.

In 1952 the US Army filed a patent on how to prepare ricin for weapon purposes [137]. Certainly the extent to which ricin was collected for military purposes is not known. It was, however, intended to be employed in assassination of Georgi Markov and Vladimir Kostov, exiled journalists who published incriminate information about the corrupt life of the Bulgarian communist leadership [1,3]. Five more instances were identified where this assassination technique was used. In the past decade ricin has been associated with terrorist organizations in several countries. The availability of improved anti-ricin vaccine [138] and better ability to trace and identify toxin in the body should make the toxin a less tempting compound for use in bioterrorism [1,139].

In 2013, CNN Justice [140] has reported that the Texas actress, Shannon G. Richardson, was sentenced to 18 years in prison after admitting last year that she sent ricin-tainted letters to the US President Barack Obama and then New York City Mayor Michael Bloomberg.

Type 1 RIPs are certainly not as cytotoxic to higher animals, since they cannot cross the cell membrane on their own [16]. Pokeweed plant synthesizes its toxin as a precursor and compartmentalizes it within cell wall matrix [15]. This ensures that the pokeweed’s ribosomes never encounter its own toxin, leaving overall protein synthesis unaffected. In contrast to the healthy appearance of pokeweed, the expression of PAP in transgenic *N. tabacum* plants leads to various physiological changes [5]. Transgenic tobacco plants producing high levels (more than 10 ng/mg protein) of PAP were sterile, having a stunted, molted phenotype. This correlated with the level of PAP expressed. Plants producing less than 1–5 ng PAP/mg protein were fertile and normal in appearance [5,134]. In recent studies, Hudak *et al.*, have shown PAP undergoing homodimerization as a mechanism to limit depurination of pokeweed ribosomes [141].

#### 2.3.3. Immunotoxins and Other Conjugates of RIPs

The first carrier-toxin hetero-conjugates of RIPs were prepared using polyclonal and later monoclonal antibodies with toxins that were able to block protein synthesis at the ribosome level. Bio-specific agents other than monoclonal antibodies (hormones, growth factors, antigens, cytokines, *etc.*) have also been employed in developing cell-targeting conjugates [142]. Toxins of different types can be used to construct effective immunotoxin (IT) conjugates, including plant, bacterial and fungal toxins. RIPs [54] have been extensively used in preparation of such ITs. These chimeric ITs can be made with either type 1 or type 2 RIPs [143]. The linkage of the carrier molecule to the toxin can be attained by chemical cross-linking, indirect linking, or gene fusion [144].

Several lines of research have efficiently used PAP as a component of ITs, conjugated to a variety of monoclonal antibodies. Jansen *et al.* [145] have used B43-PAP immunotoxin plus cyclophosphamide to successfully treat human leukemia in mice with severe combined immunodeficiency (SCID). Uckun *et al.*, have used B43 (anti-CD19)-PAP IT in treatment of human pre-B acute lymphoblastic and other types of leukemia in mice [146,147,148,149]. Erice *et al.* [150] have found that PAP conjugated to monoclonal antibodies recognizing CD4, CD5, or CD7 antigens effectively inhibited HIV-1 replication in normal CD4+ T cells infected with HIV-1 strain LAVBRU, as well as in activated T cells from two asymptomatic HIV-1-seropositive individuals [151]. All of the above and many other lines of evidence point toward potential therapeutic PAP-immunoconjugate applications of this protein against a variety of cancer lines as well as HIV-1.

Liposomal delivery of RIPs may provide both hydrophilic, hydrophobic environments, enhancing RIP solubility. It uses regulated drug release, and thus reduces or eliminates tissue damage on accidental extravasation, and protects RIP from premature degradation, functions as a sustained release system, and can substantially alter the pharmacokinetics of the RIP and reduce clearance [152]. As all of the above properties of targeted liposomal employment of RIPs become surmounted, RIP-conjugated immunotoxins may become an important new modality for cancer therapy. The major dose-limiting toxicity of RIP-conjugated immunotoxin therapies is vascular leak syndrome (VLS) [153]. VLS is characterized by an increase in vascular permeability accompanied by extravasation of fluids and proteins resulting in interstitial edema and organ failure.

#### 2.3.4. Interactions of PAP with VPg and the Inhibition of PAP Antiviral Activity

Researchers have undertaken extensive efforts in exploration of RIP properties and studies of their toxicities in order to develop antidotes against their activity. Biochemical and structural characterization of the catalytic domains of many RIPs, including RTA, served as an attractive target for structure-based drug design. Our comprehension of the RIP action mechanism predominantly comes from the structural and mutagenesis work [154,155]. Advances in the X-ray crystallography allowed for the determination of the high-resolution structures of RIP catalytic domains, and thus development of substrate analogues that possess high affinity for the RIP active sites. Several categories of RIP inhibitors have been developed. Thus far, effective small molecule RTA inhibitors are generally based on pterins, guanines, pyrimidines, and stem-loop oligonucleotides. Schramm and his colleagues have developed transition state analogues that have high affinities for the RTA active site; these however, only bind at acidic pH, around 4 [97,98]. In recent years, RIP activity of PAP was shown to be inhibited by a viral peptide—genome-linked protein, VPg isolated from the turnip mosaic potyvirus (TuMV) [107]. High affinity of the viral peptide for PAP (*K*_d_ = 29 nM), and its ability to inhibit PAP enzymatic activity, provides a new direction in search for a novel generation of RIP inhibitors.

The genus *Potyvirus* contains more than 200 members and belongs to one of the largest plant virus family—*Potyviridae* [156]. Potyviruses contain an approximately 10 kilobases positive-sense ssRNA that is covalently linked to a viral genome-linked protein (VPg) at their 5' end via a tyrosine residue [157] and polyadenylated at the 3'end [158,159,160]. The RNA has a single open reading frame that is translated into a large polyprotein. The potyviral polyprotein is proteolytically processed into mature proteins by dedicated virus-encoded proteases [161]. It has been suggested that VPg may serve as an analogue of the m^7^G cap of the mRNAs, and plays a role in mRNA translation because of its interactions with the cap-binding eIF4E (eIFiso4E) and eIF4F (eIFiso4F) proteins [162,163]. Studies also support a biological role for the VPg linked to the viral RNA in virions. VPg is necessary for the infectivity of the virus [164], cell-to-cell movement [165,166,167,168,169,170], and has been linked to a variety of other viral necessities.

Interactions between PAP and VPg were studied over a range of different temperatures using direct fluorescence titrations. These interactions were recognized as competitive [107], because VPg competed with the TEV RNA for PAP binding. Thermodynamics of the PAP-VPg binding were identified as enthalpically driven and entropically favorable [107], and exhibited similarities to those of eIF*iso*4E- and eIF*iso*4F-VPg binding [163]. Nearly one-third contribution from the *T*Δ*S* van’t Hoff component to the overall energy suggests that these interactions are driven by structural changes in both proteins, in a way where hydrophobic residues become less solvent exposed. In addition, PAP showed greater affinity for the viral peptide, as compared to m^7^GTP-cap analogue [121] and eIF*iso*4F [163]. Greater affinity of PAP for VPg than that for the cap structure would produce an advantage for the cell if VPg were to localize PAP to viral RNA for depurination. Beguilingly, VPg inhibited PAP activity by decreasing the mounts of purines released from various RNAs [107], suggesting that it may participate in viral strategy to overcome one of the potential host cell defense mechanisms. This is further supported Baldwin *et al.* [121], and conforms to the accepted function of PAP as a RIP. Although there are suggested similarities in the thermodynamics of PAP-VPg interactions with the eIF binding, different equilibrium dissociation constant values point toward the differences in the active sites of PAP and the cap-binding sites of the initiation factors.

## 4. Conclusions

Exploitation of RIPs as potential targets in bioterrorism, and their usage as possible antiviral and anti-cancer agents deserve further attention. Cytotoxicity of RIPs and their effects on biological systems present the investigators with novel ideas in exploration of new pathways for the inhibition of RIP activity, as well as modulation of the current inhibitors to perfect their action. These inhibitors may even assist in controlling non-specific cytotoxicity of RIP-immunoconjugates, and serve as antidotes against their toxicity. Recently, there has been an interest in structure-based drug design that uses the knowledge of protein structure and its ligand interactions to identify potent enzyme inhibitors. The X-ray crystal structures of ricin, PAP and other RIPs have been solved, and the presence of various substrate analogs interacting with RTA side chain amino acids has been mapped out [28,30,171], identifying pivotal residues for catalysis [172]. Kurinov *et al.*, have reported crystal structures of PAP co-crystallized with adenyl-guanosine (ApG) and adenyl-cytosine-cytosine (ApCpC), and showed evidence for a broad spectrum *N*-glycosidase activity of PAP toward adenine-containing single stranded RNA [34]. The inhibitory action of VPg on RIPs has not been studied in great detail, and may present researchers with new insights in understanding not only the inhibitory mechanism of PAP and other RIPs, but also a deeper insight into an evolutionary adaptation of plant-virus interactions, and a new direction in understanding how these plant-pathogen relations have shaped each other for generations. Khan *et al.*, have shown that VPg may serve as a cap analog [162,163], and stimulates the *in vitro* translation of uncapped IRES-containing RNA to promote viral gene translation [173]. In addition, VPg inhibits cellular capped RNA translation in wheat germ extract by recruiting the translation initiation factors (4E and *iso*4E) [173]. The central domain of potyviral VPg is involved in the interactions with the eIF [174], and alterations within VPg structure abolish these interactions [159]. The *N*-terminal truncation renders VPg unable to interact with the eIF4E and eIF*iso*4E [159], and the binding site on VPg for the eIFs overlaps with that for PAP binding [107]. PAP may therefore have antiviral activity both through ribosome inactivation and RNA depurination as well as binding to VPg and potentially sequestering it. VPg is essential for viral replication and this may be a new role for PAP in antiviral defense. Future investigations of the inhibitory effects of VPg on other RIP activity may provide researchers with a novel and natural peptide inhibitors of the cytotoxic activity of RIPs. This novel peptide inhibitor may aid in non-specific inhibition of RIP activity when used as immunoconjugates in anti-cancer of anti-viral regiments. RIP immunotoxins constitute a new modality for the treatment of cancer, since they target cells displaying specific surface-receptors and antigens. These chimeric proteins consisting of an antibody linked to a toxin. The antibody confers specificity (ability to recognize and react with the target), whereas the toxin confers cytotoxicity (ability to kill the target cell) [175,176]. Immunotoxins have been used in both mice and humans to eliminate tumor cells, autoimmune cells, and virus-infected cells [177,178,179]. For instance, Uckun *et al.*, have successfully used PAP conjugated to TP-3, an IgG2b mAb that recognizes human and canine osteosarcomas and budding capillaries of tumors, to lower the viability of human OHS osteosarcoma cells [180,181]. However, many obstacles arise from using RIPs as immunotoxins. Examples include, but not limited to, tissue damage on extravasation, rapid breakdown *in vivo*, unfavorable pharmacokinetics, poor biodistribution, and lack of selectivity for target tissues [152,181,182]. Immunoconjugates encapsulated into liposomes may alleviate these problems, and have been successfully used in the past for treatment of many types of cancers [183,184]. Immunoconjugated liposomal PAP may provide a new and promising direction in cancer therapeutics, and alleviate the above obstacles in cancer treatments.
